# Rhizobia with 16S rRNA and *nifH* Similar to *Mesorhizobium huakuii* but Novel *recA*, *glnII*, *nodA and nodC* Genes Are Symbionts of New Zealand *Carmichaelinae*

**DOI:** 10.1371/journal.pone.0047677

**Published:** 2012-10-31

**Authors:** Heng Wee Tan, Bevan S. Weir, Noel Carter, Peter B. Heenan, Hayley J. Ridgway, Euan K. James, Janet I. Sprent, J. Peter W. Young, Mitchell Andrews

**Affiliations:** 1 Faculty of Agriculture and Life Sciences, Lincoln University, Christchurch, New Zealand; 2 Systematics Group, Landcare Research, Auckland, New Zealand; 3 Faculty of Applied Sciences, University of Sunderland, Sunderland, United Kingdom; 4 Allan Herbarium, Landcare Research, Lincoln, Christchurch, New Zealand; 5 Ecological Sciences, James Hutton Institute, Invergowrie, Dundee, United Kingdom; 6 College of Life Sciences, University of Dundee, Dundee, United Kingdom; 7 Department of Biology, University of York, York, United Kingdom; Dowling College, United States of America

## Abstract

New Zealand became geographically isolated about 80 million years ago and this separation gave rise to a unique native flora including four genera of legume, *Carmichaelia*, *Clianthus* and *Montigena* in the Carmichaelinae clade, tribe Galegeae, and *Sophora*, tribe Sophoreae, sub-family Papilionoideae. Ten bacterial strains isolated from NZ Carmichaelinae growing in natural ecosystems grouped close to the *Mesorhizobium huakuii* type strain in relation to their 16S rRNA and *nifH* gene sequences. However, the ten strains separated into four groups on the basis of their *recA* and *glnII* sequences: all groups were clearly distinct from all *Mesorhizobium* type strains. The ten strains separated into two groups on the basis of their *nodA* sequences but grouped closely together in relation to *nodC* sequences; all *nodA* and *nodC* sequences were novel. Seven strains selected and the *M. huakuii* type strain (isolated from *Astragalus sinicus*) produced functional nodules on *Carmichaelia* spp., *Clianthus puniceus* and *A. sinicus* but did not nodulate two *Sophora* species. We conclude that rhizobia closely related to *M. huakuii* on the basis of 16S rRNA and *nifH* gene sequences, but with variable *recA* and *glnII* genes and novel *nodA* and *nodC* genes, are common symbionts of NZ *Carmichaelinae*.

## Introduction

Members of the Fabaceae (the legume family) are components of most of the world's vegetation types [1]. Many legume species have the capacity to fix atmospheric nitrogen (N_2_) via symbiotic bacteria (generally termed ‘rhizobia’) in root nodules and this can give them an advantage under low soil N conditions if other factors are suitable for growth [2], [3], [4]. New Zealand (NZ) became geographically isolated about 80 million years ago [5], [6] and this separation gave rise to a unique native flora [7] that is considered to have evolved during the late Cenozoic [8], [9]. There are four genera of legume, of the sub-family Papilionoideae on the main New Zealand islands, plus the strand species *Canavalia rosea* which occurs in the Kermadec Islands [10]. The four genera are the closely related *Carmichaelia* (23 endemic species), *Clianthus* (2 endemic species) and *Montigena* (1 endemic species) in the Carmichaelinae clade, tribe Galegeae, and *Sophora* (8 endemic species), within the tribe Sophoreae [11], [12], [13], [14], [15]. All species in all four genera are capable of nodulation, but genotypic data on the rhizobia which induce nodules on these plants are limited [4], [16].

Previously, sequences were obtained for the small subunit ribosomal RNA (16S rRNA) gene of twenty bacterial strains isolated from species of the four NZ native legume genera growing in natural ecosystems [16]. Most isolates aligned with the genus *Mesorhizobium* either as named species or as putative novel species. Five strains from *Carmichaelia*, and one each from *Clianthus* and *Montigena*, were most closely related to *Mesorhizobium huakuii* (*M. huakuii*). However, this study did not assess the ability of the isolated rhizobial strains to nodulate NZ native legumes or sequence any of their N_2_-fixation (*nif*) or nodulation (*nod*) genes. A separate study, characterised two bacterial strains, Ca004 and Cc3, isolated from *Carmichaelia australis* and *Carmichaelia corrugata* respectively, growing in natural ecosystems, using 16S rRNA sequences [17]. Both strains aligned closely with *M. huakuii* and produced functional nodules on the five *Carmichaelia* spp. tested. In the current study, six strains isolated from *Carmichaelia* spp. and *Montigena* aligned with *M. huakuii* on the basis of their 16S rRNA sequences.

Here we focus on bacterial strains isolated from NZ native legumes growing in natural ecosystems in the current and previous studies which aligned closely with *M. huakuii* on the basis of their 16S rRNA sequences. In addition to 16S rRNA, selected ‘housekeeping’ and *nif* and *nod* genes were sequenced from the strains and their ability, and that of the *M. huakuii* type strain originally isolated from *Astragalus sinicus* [18], [19], to nodulate species of the different NZ native legume genera and *A. sinicus* is assessed.

## Materials and Methods

### Bacterial strains and culture media

Ten bacterial strains isolated from species in the Carmichaelinae growing in natural ecosystems in the current and previous studies were examined. All strains are deposited in the International Collection of Microorganisms from Plants (ICMP), Landcare Research, Auckland, NZ. Strains ICMP 18942, ICMP 18943 and ICMP 19420 from *Montigena novae-zelandiae* (Mn) and ICMP 19041, ICMP 19042 and ICMP 19043 from *C. australis* (Ca), *C. monroi* (Cm) and *C. nana* (Cn) respectively were isolated in the current study from plants sampled at Dry Stream, Torlesse Range, Canterbury, NZ (43°16′S 171°43′E) in April 2010. All necessary permits for the described field studies were obtained from the Department of Conservation, NZ. Christine Fernyhough and John Bougen, owners of Castle Hill Station, are thanked for permission to collect plants, seed and rhizobia from Dry Stream. Strains ICMP 11541, ICMP 12690 and ICMP 13190 isolated previously from *Clianthus puniceus* (Clp), *Montigena novae-zelandiae* and *C. australis* respectively [16] and the *M. huakuii* type strain (ICMP 11069) were obtained from the ICMP collection directly. Strain ICMP 19418 ( = strain Ca004) [17] was sourced from the University of York rhizobium collection.

For strains isolated in the current study, root nodules were surface sterilised in a laminar flow cabinet by immersion in 96% ethanol for 5 seconds and 5% sodium hypochlorite for 3 minutes then rinsed with sterile water. Surface sterilised nodules were squashed in sterile water then this suspension was streaked onto a yeast mannitol agar (YMA) [20] plate and incubated at 20–25°C in the dark for 2–4 days. A purified culture or a single colony by sub-culture was obtained from each plate. Samples of these cultures and those from the ICMP collection were inoculated into a suspension of Yeast Mannitol Broth (YMB) [20] and used for preparation of subcultures for DNA extraction or inoculum.

### Sequencing of the 16S rRNA, housekeeping and symbiotic genes

DNA was extracted from the bacterial cultures using the standard Qiagen-Gentra PUREGENE DNA Purification Kit for gram-negative bacteria. Six genes were studied: 16S rRNA, DNA recombinase A (*recA*), glutamine synthetase II (*glnII*), nitrogenase iron protein (*nifH*), N-acyltransferase nodulation protein A (*nodA*) and N-acetylglucosaminyl transferase nodulation protein C (*nodC*). Primers for PCR amplification with their sequences and sources are shown in Table 1. All primers were manufactured by Integrated DNA Technologies, Auckland, NZ. All PCR amplifications were performed using the FastStart™ Taq DNA Polymerase kit (Roche Applied Science, Auckland) optimised for annealing temperature and primer concentration, if required. The PCR products were resolved via gel electrophoresis (1% agarose gel in 1×Tris-acetate-EDTA buffer) followed by staining with ethidium bromide solution and viewing under UV light. PCR products were sequenced by the Bio-Protection Research Centre Sequencing Facility, Lincoln University, Lincoln, NZ and DNA sequence data were obtained via Sequence Scanner v 1.0 software (©Applied Biosystems) and edited and assembled using DNAMAN Version 6 (©Lynnon Biosoft Corporation).

**Table 1:** Oligonucleotide primers used in this study.

Target gene	Primer	Sequence (5′-3′)*	Reference
16S rRNA	F27	AGA-GTT-TGA-TCM-TGG-CTC-AG	[21]
	FGPS485F	CAG-CAG-CCG-CGG-TAA	[22]
	R1494	CTA-CGG-YTA-CCT-TGT-TAC-GAC	[21]
*recA*	41F	TTC-GGC-AAG-GGM-TCG-RTS-ATG	[23]
	640R	ACA-TSA-CRC-CGA-TCT-TCA-TGC	
*glnII*	GSll-1	AAC-GCA-GAT-CAA-GGA-ATT-CG	[24]
	GSll-2	ATG-CCC-GAG-CCG-TTC-CAG-TC	
	GSll-3	AGR-TYT-TCG-GCA-AGG-GYT-C	
	GSll-4	GCG-AAC-GAT-CTG-GTA-GGG-GT	
*nifH*	PolF	TGC-GAY-CCS-AAR-GCB-GAC-TC	[25]
	PolR	ATS-GCC-ATC-ATY-TCR-CCG-GA	
*nodC*	α-nodCF	AYG-THG-TYG-AYG-ACG-GTT-C	[26]
	α-nodCR	CGY-GAC-AGC-CAN-TCK-CTA-TTG	
*nodA*	nodA1	TGC-RGT-GGA-ARN-TRN-NCT-GGG-AAA	[27]
	nodA3	TCA-TAG-CTC-YGR-ACC-GTT-CCG	[28]

* A, C, G, T = standard nucleotides; M = C or A; Y = C or T; R = A or G; S = G or C; B = T or C or G; H = A or C or T; N = A or G or C or T; K = T or G.

### Phylogenetic analyses

DNA sequences were aligned and Maximum Likelihood trees constructed with 500 bootstrap replications with partial deletion and an 80% coverage cut off using MEGA5 software [29]. The most closely related *Mesorhizobium* type strains and strains isolated from NZ native legumes which were closely related to *M. huakuii* on the basis of 16S rRNA and available on the Genbank sequence database (www.ncbi.nlm.nih.gov/genbank) were used for the 16S rRNA, *recA*, *glnII*, *nifH*, *nodA* and *nodC* trees. For the *nodA* tree, we also included the sequence from a strain (TM1) recently isolated from *Thermopsis lupinoides* and characterised as *M. huakuii* on the basis of 16S rRNA [30]. For the *nodC* tree, strains characterised as *M. huakuii* on the basis of their 16S rRNA sequences were used as there is no *nodC* sequence for the *M. huakuii* type strain in Genbank and we were unable to sequence it. MEGA5 model test was performed to select a model of nucleotide substitution and the ‘best’ model (lowest Bayesian Information Criterion (BIC) score) used for each gene. The Kimura 2-parameter (K2), gamma distribution (+G) with invariant sites (+I) model was used for 16S rRNA. The Tamura 3-parameter (T92), gamma distribution (+G) model was used for all other genes. Only bootstrap probability values ≥50% are shown. The sequences obtained in this study have been deposited in the GenBank sequence database and their accession numbers are shown in the figures.

### Nodulation and N_2_ fixation studies

Seven bacterial strains (ICMP 12690 (Mn), ICMP 18942 (Mn), ICMP 13190 (Ca), ICMP 19041 (Ca), ICMP 19042 (Cm), ICMP 19418 (Ca) and ICMP 11541 (Clp)) were selected for nodulation and N_2_ fixation studies. Seeds of *Carmichaelia* spp. and *M. novae-zelandiae* were collected from the field site (Dry Stream, Torlesse Range, Canterbury); seeds of *Sophora* spp. and *Clianthus puniceus* were purchased from New Zealand Tree Seeds, Rangiora, NZ and seeds of *Astragalus sinicus* obtained from the Margot Forde Germplasm Centre, Palmerston North, NZ. All plant procedures carried out until processing of nodules, took place under sterile conditions. All seeds were, in sequence, soaked in concentrated sulphuric acid for 30–90 minutes, rinsed with sterile water then soaked in hot (∼60°C) sterile water which was left at room temperature overnight. Seeds were then transferred to water agar plates. After germination, seedlings were transferred to polyethylene terephthalate jars (two seedlings per jar) containing vermiculite and supplied a complete nutrient medium (pH 6.0) containing NH_4_NO_3_ (0.1 mM), CaCl_2_ (1.0 mM), KCl (1.0 mM), MgSO_4_.7H_2_O (1.0 mM), NaH_2_PO_4_ (1.0 mM), Na_2_HPO_4_ (0.1 mM), FeCl_2_.4H_2_O (5.0 µM), H_3_BO_3_ (5.0 µM), MnCl_2_.2H_2_O (1.0 µM), Na_2_MoO_4_.2H_2_O (0.5 µM), CuSO_4_.5H_2_O (0.1 µM), ZnSO_4_.7H_2_O (0.1 µM) and CoCl_2_.6H_2_O (0.02 µM). Plants were grown in a controlled environment cabinet and exposed to a 16 h photoperiod (400 µmol photons m^−2^ s^−1^) at a constant 22°C.

At 5–15 days after sowing, seedlings were inoculated with the appropriate rhizobial strain grown to log phase: uninoculated plants supplied YMB only were used as controls. There were at least 3 replicate jars per treatment. Plants were inspected at two weekly intervals for nodulation and at 30–50 days after inoculation were tested for nitrogenase activity using the acetylene reduction assay (ARA) [31]. After the ARA, rhizobial strains were isolated from three to six nodules per treatment and their 16S rRNA gene sequenced.

## Results

**Figure 1:**
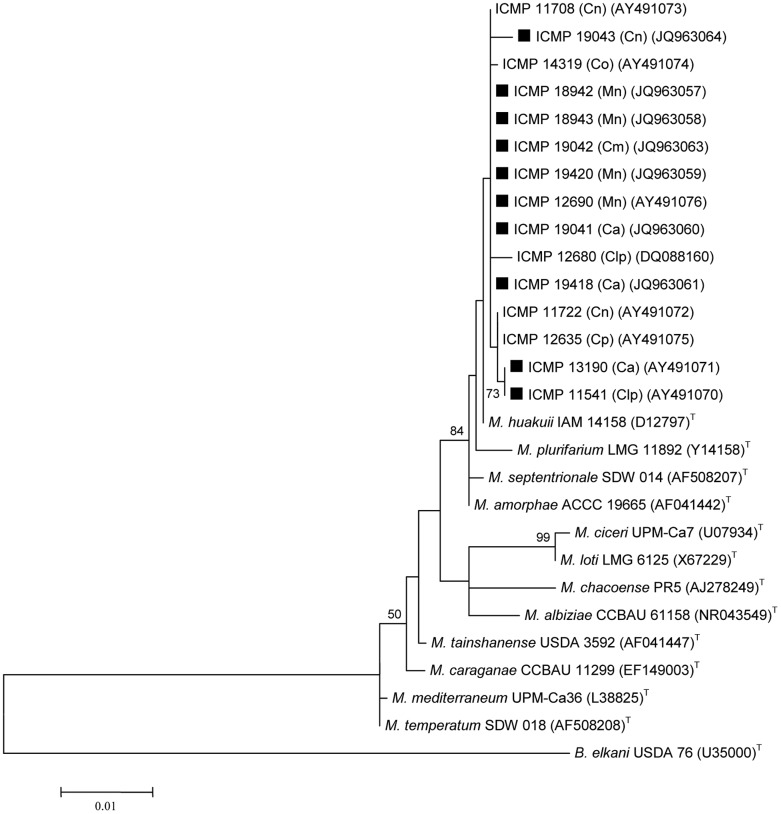
16S rRNA gene phylogenetic tree of bacterial strains isolated from New Zealand native legumes and selected *Mesorhizobium* type strains. Ca = *Carmichaelia australis*; Cm = *Carmichaelia monroi*; Cn = *Carmichaelia nana*; Co = *Carmichaelia odorata*; Cp = *Carmichaelia petriei*; Clp = *Clianthus puniceus*; Mn = *Montigena novae-zelandiae*. **▪** indicates strains focussed on in current study. Numbers on branches are bootstrap % from 500 replicates (shown only when ≥50%). The tree was rooted with the *Bradyrhizobium elkani* type strain.

All ten bacterial strains isolated from *Carmichaelia*, *Clianthus* or *Montigena* studied here clustered closely with the *M. huakuii* type strain and five other strains previously isolated from *Carmichaelia* or *Clianthus* spp. (ICMP 11708, ICMP 14319, ICMP 12680, ICMP 11722 and ICMP 12635) [16], [32], on the basis of their 16S rRNA sequences (Fig. 1). Eight strains, ICMP 18942, ICMP 18943, ICMP 19042, ICMP 19420, ICMP 12690, ICMP 19041 and ICMP 19418 this study and ICMP 11708 were identical and showed 99.5% similarity (1200 bp) to the *M. huakuii* type strain.

**Figure 2:**
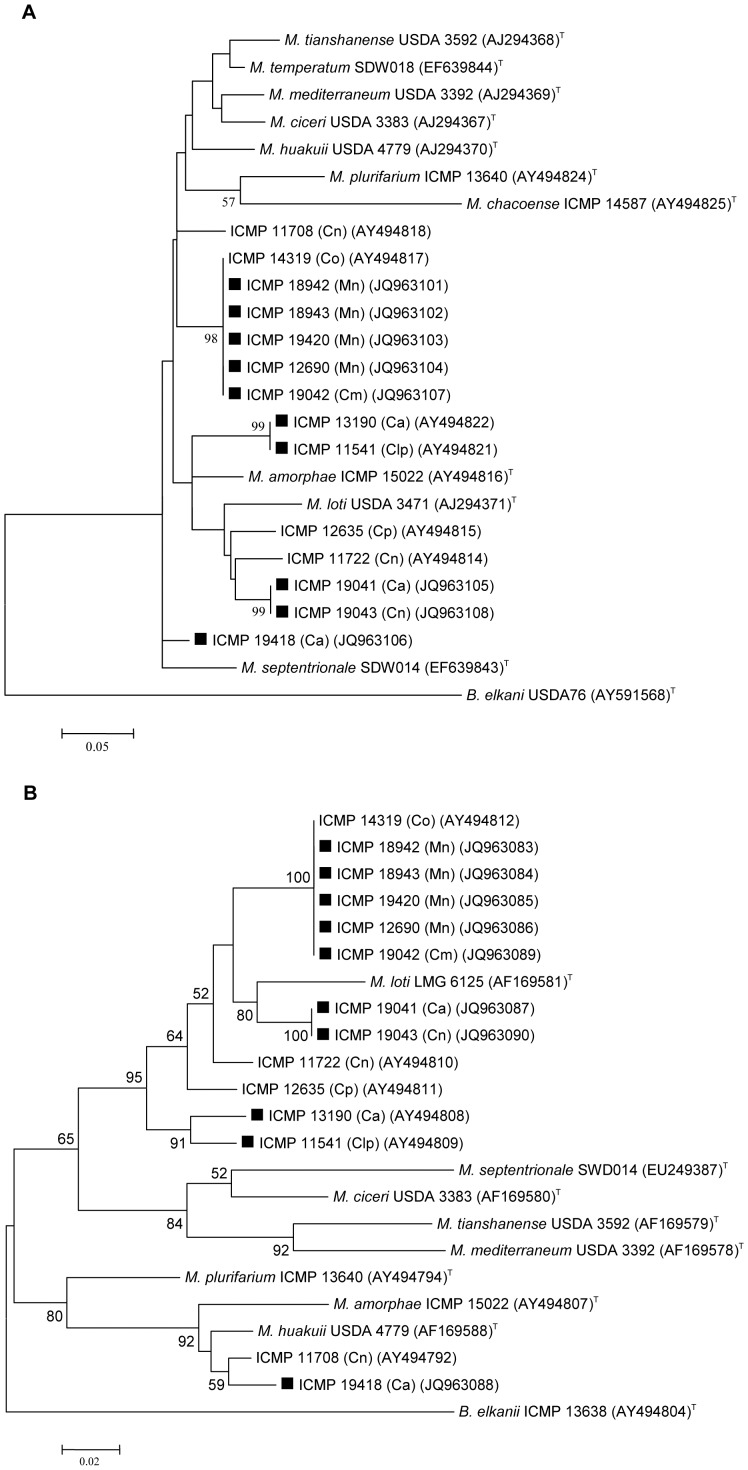
*recA* (a) and *glnII* (b) gene phylogenetic trees of bacterial strains isolated from New Zealand native legumes and selected *Mesorhizobium* type strains. Ca = *Carmichaelia australis*; Cm = *Carmichaelia monroi*; Cn = *Carmichaelia nana*; Co = *Carmichaelia odorata*; Cp = *Carmichaelia petriei*; Clp = *Clianthus puniceus*; Mn = *Montigena novae-zelandiae*. **▪** indicates strains focussed on in this study. Numbers on branches are bootstrap % from 500 replicates (shown only when ≥50%). The trees were rooted with the *Bradyrhizobium elkani* type strain.

The ten strains separated into four groups on the basis of their *recA* sequences with all four groups clearly separated from all *Mesorhizobium* type strains (Fig. 2a). The four groupings on the basis of the *recA* sequences held in relation to *glnII* sequences (Fig. 2b). For *glnII* sequences, three groups (nine strains) were most closely related to, but clearly separated from, the *Mesorhizobium loti* type strain while strain ICMP 19418 (Ca) was most closely related to *M. huakuii*. Three other strains previously isolated from *Carmichaelia* spp. (ICMP 14319, ICMP 11722 and ICMP 12635) [16] also aligned closest to *M. loti*.

**Figure 3:**
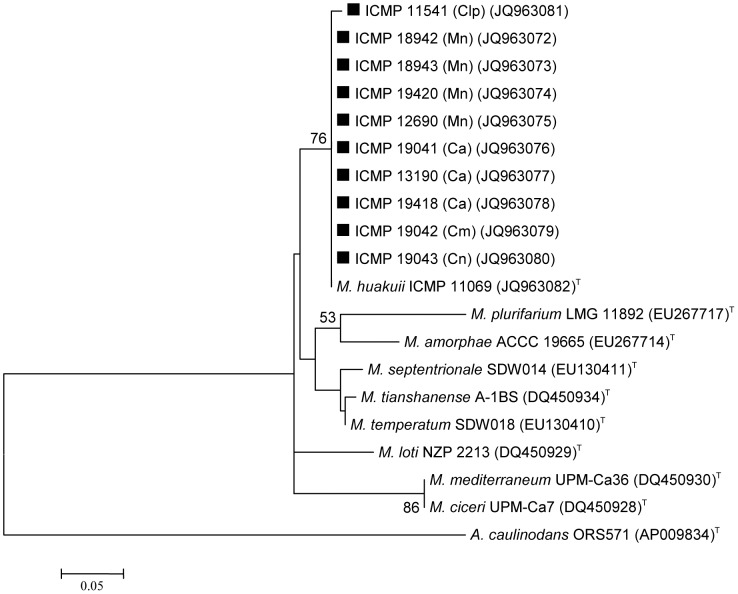
*nifH* gene phylogenetic tree of bacterial strains isolated from New Zealand native legumes and selected *Mesorhizobium* type strains. Ca = *Carmichaelia australis*; Cm = *Carmichaelia monroi*; Cn = *Carmichaelia nana*; Clp = *Clianthus puniceus*; Mn = *Montigena novae-zelandiae*. **▪** indicates strains focussed on in this study. Numbers on branches are boot strap % from 500 replicates (shown only when ≥50%).

**Figure 4:**
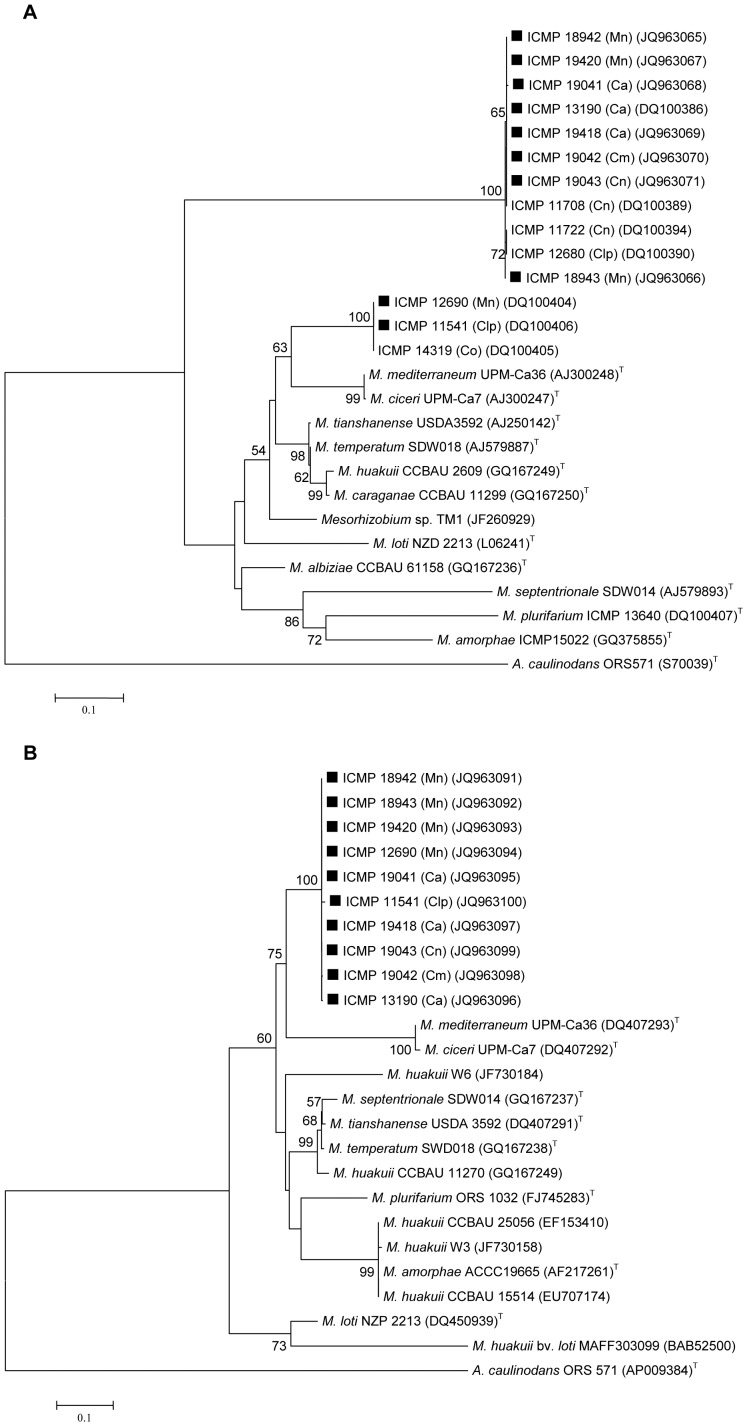
*nodA* (a) and *nodC* (b) gene phylogenetic trees of bacterial strains isolated from New Zealand native legumes, selected *Mesorhizobium* type strains and strains characterised as *Mesorhizobium huakuii* on the basis of their 16S rRNA sequences. Ca = *Carmichaelia australis*; Cm = *Carmichaelia monroi*; Cn = *Carmichaelia nana*; Co = *Carmichaelia odorata*; Clp = *Clianthus puniceus*; Mn = *Montigena novae-zelandiae*. **▪** indicates strains focussed on in this study. Numbers on branches are bootstrap % from 500 replicates (shown only when ≥50%).

There was little variation in the *nifH* gene sequences across the ten strains studied (Fig. 3). With the exception of ICMP 11541 (Clp), all strains were identical (290 bp) to the *M. huakuii* type strain (sequenced in the current study). The ten strains separated into two groups on the basis of their *nodA* sequences (Fig. 4a). The larger group (eight strains) clustered with three other strains (ICMP 11708, ICMP 11722 and ICMP 12680) previously isolated from *Carmichaelia* spp. or *Clianthus puniceus* [32], while the smaller group (two strains) grouped with strain ICMP 14319 isolated from *Carmichaelia odorata* [32]. The *nodA* sequences for the two groups showed only 66.29% similarity (530 bp) to each other but both separated clearly from all *Mesorhizobium* type strains and strain TMI isolated from *Thermopsis lupinoides* and characterised as *M. huakuii* on the basis of its 16S rRNA sequence [30].

All ten strains grouped closely together in relation to their *nodC* sequences and as for *nodA* sequences separated from all others on the Genbank database including those of strains characterised as *M. huakuii* on the basis of their 16S rRNA sequences (Fig. 4b) [33], [34].

All seven Carmichaelinae strains tested and the *M. huakuii* type strain produced functional nodules on *Carmichaelia australis*, *Carmichaelia kirkii*, *Carmichaelia petriei*, *Clianthus puniceus* and *Astragalus sinicus* but did not nodulate either of the two *Sophora* species (Table 2). In all cases, the 16S rRNA sequence for the strain recovered from nodules after the acetylene reduction assay was identical to that of the strain used as inoculant.

**Table 2:** Host specificity of rhizobial strains used in this study.

	Species tested						
	*Carmichaelia*			*Sophora*		*Clianthus*	*Astragalus*
Strain	*australis*	*kirkii*	*petriei*	*microphylla*	*tetraptera*	*puniceus*	*sinicus*
ICMP 18942 (Mn*)	Nod+Fix+	Nod+Fix+	Nod+Fix+	Nod−	Nod−	Nod+Fix+	Nod+Fix+
ICMP 12690 (Mn)	Nod+Fix+	Nod+Fix+	Nod+Fix+	Nod−	Nod−	Nod+Fix+	Nod+Fix+
ICMP 19041 (Ca)	Nod+Fix+	Nod+Fix+	Nod+Fix+	Nod−	Nod−	Nod+Fix+	Nod+Fix+
ICMP 19042 (Cm)	Nod+Fix+	Nod+Fix+	Nod+Fix+	Nod−	Nod−	Nod+Fix+	Nod+Fix+
ICMP 19418 (Ca)	Nod+Fix+	Nod+Fix+	Nod+Fix+	Nod−	Nod−	Nod+Fix+	Nod+Fix+
ICMP 13190 (Ca)	Nod+Fix+	Nod+Fix+	Nod+Fix+	Nod−	Nod−	Nod+Fix+	Nod+Fix+
ICMP 11541 (Clp)	Nod+Fix+	Nod+Fix+	Nod+Fix+	Nod−	Nod−	Nod+Fix+	Nod+Fix+
*M. huakuii*	Nod+Fix+	Nod+Fix+	Nod+Fix+	Nod−	Nod−	Nod+Fix+	Nod+Fix+

* Original host: Mn = *Montigena novae-zelandiae*; Ca = *Carmichaelia australis*; Cm = *Carmichaelia monroi*; Clp = *Clianthus puniceus*.

Nod− = no plants nodulated; Nod+ = all plants nodulated; Fix+ = nitrogen fixing nodules.

## Discussion

Genotypic data on the rhizobia which induce nodules on NZ native legumes are limited but it seems likely that rhizobial symbionts co-evolved with NZ native legumes in isolation from the regions of major legume evolution and as a result, may have novel characteristics. Here we focus on ten bacterial strains isolated from NZ native legumes in the current and previous studies which closely aligned with *M. huakuii* on the basis of their 16S rRNA sequences. It is shown that the ten strains also grouped closely to the *M. huakuii* type strain in relation to their *nifH* sequences. However, with the exception of *glnII* for strain ICMP 19418 (Ca), all sequences for *recA*, *glnII*, *nodA* and *nodC* were substantially different for the ten strains and the *M. huakuii* type strain. The ten strains separated into the same four groups on the basis of their *recA* and *glnII* sequences. For *recA*, the four groups clearly separated from all *Mesorhizobium* type strains. For *glnII*, three groups (nine strains) aligned closest to but clearly separated from the *M. loti* type strain. It is possible that *Mesorhizobium* evolved in NZ with variable *recA* and *glnII* genes. However, many legume species have been introduced and have naturalised in NZ since colonisation by Europeans in the 19^th^ Century. Also, in the case of crop legumes, rhizobial inoculant, was often applied, and in some cases still is, and it has been shown that chromosomal symbiosis genes can transfer (‘lateral’ transfer) from a *Mesorhizobium loti* inoculant strain to indigenous *M. huakuii* strains in NZ soils [35]. Thus, the possibility of genetic exchange of *recA* and *glnII* genes between recently introduced rhizobia and the indigenous *Mesorhizobium* should be considered. Certainly, there is strong evidence that in the past, horizontal transfer of the *glnII* gene has occurred between major rhizobial groups [24], [36], but it would require substantial further work to determine if this has happened between rhizobia in NZ over the past 150 years.

All the strains grouped closely together in relation to their *nodC* sequences and these sequences separated from all others in Genbank. There was no *nodC* sequence for the *M. huakuii* type strain in Genbank and we were unable to sequence it. However, the *nodC* sequences for the strains separated from those of other strains characterised as *M. huakuii* on the basis of their 16S rRNA sequences. The ten strains separated into two groups in relation to their *nodA* sequences, eight of the strains studied grouped with three other strains previously isolated from *Carmichaelia* spp. or *Clianthus puniceus* while two strains grouped with a strain isolated from *Carmichaelia odorata* [32]. The *nodA* sequences for both groups separated clearly from those of all the *Mesorhizobium* type strains and the ‘novel’ *nodA* sequence reported for the *M. huakuii* strain isolated from *Thermopsis lupinoides* in Kamtchatka, Russia [30]. Thus, although the *nifH* gene of the ten strains aligns closely with that of *M. huakuii*, their *nodC* and *nodA* genes are novel, and it seems likely that they evolved in rhizobia indigenous to NZ.

All seven strains selected (isolated from three separate genera, over three separate studies and including isolates from the North and South Islands of NZ) produced functional nodules on the three *Carmichaelia* spp. and *Clianthus puniceus* tested indicating that this group of bacteria are common rhizobial symbionts of Carmichaelinae species in NZ. None of the strains produced functional nodules on the *Sophora* spp. tested indicating that, within NZ native legumes, they are specific to Carmichaelinae species. Host range in rhizobia is at least in part determined by the structure of the lipo-chitin oligosaccharides ‘nod factors’ synthesised by the products of nodulation genes such as *nodA* and *nodC* [31], [37]. Thus, it is to some extent unexpected that, although the *nodA* and *nodC* genes of the strains are novel, all seven strains tested, produced functional nodules on *Astragalus sinicus*, and that the *M. huakuii* type strain isolated from *A. sinicus* produced functional nodules on the *Carmichaelia* spp. and *Clianthus puniceus*. As the strains form nodules on *Astragalus sinicus*, they do not meet the criteria required to be considered as a different *M. huakuii* symbiovar [38].

Four of the strains isolated and characterised in this study (ICMP 18942 (Mn), ICMP 18943 (Mn), ICMP 19420 (Mn) and ICMP 19042 (Cm)) were obtained from plants growing in alpine scree and rock bluff habitats that have not been utilised for agricultural purposes. The 16S rRNA, and *nifH* sequences for these strains aligned closest to *M. huakuii*, their *glnII* sequence aligned closest to but separate from that of *M. loti* and they had novel *recA*, *nodA* and *nodC* genes. We suggest that these strains are indigenous NZ strains that evolved with their hosts *Carmichaelia* and *Montigena* during the late-Miocene, as these two legume genera diverged from a common ancestor about 5 mya [39]. Overall, we conclude that rhizobia closely related to *M. huakuii* on the basis of 16S rRNA and *nifH* gene sequences, but with variable *recA* and *glnII* genes and novel *nodA* and *nodC* genes, are common symbionts of NZ Carmichaelinae.

## References

[1] Lewis G, Schrire B, Mackinder B, Lock M (2005) Legumes of the world. London: Royal Botanic Gardens.

[2] Andrews M, Lea PJ, Raven JA, Azevedo RA (2009) Nitrogen use efficiency. 3. Nitrogen fixation. Genes and costs. Ann Appl Biol 155: 1–13.

[3] Andrews M, James EK, Sprent JI, Boddey RM, Gross E, et al (2011) Nitrogen fixation in legumes and actinorhizal plants in natural ecosystems: values obtained using ^15^N natural abundance. Plant Ecology & Diversity 4: 131–140.

[4] Sprent JI (2009) Legume Nodulation A Global Perspective. Chichester: Wiley-Blackwell.

[5] Stevens GR (1985) Lands in Collision: Discovering New Zealand's Past Geography. Wellington: DSIR Publishing Centre.

[6] Stevens G, McGlone M, McCulloch B (1988) Prehistoric New Zealand. Auckland: Heinemann Reed.

[7] McGlone MS, Duncan RP, Heenan PB (2001) Endemism, species selection and the origin and distribution of the vascular plant flora of New Zealand. J Biogeogr 28: 199–216.

[8] Pole M (1994) The New Zealand flora – entirely long-distance dispersal? J Biogeogr 21: 625–635.

[9] Landis CA, Campbell HJ, Begg JG, Mildenhall DC, Paterson AM, et al (2008) The Waipounamu Erosion Surface: questioning the antiquity of the New Zealand land surface and terrestrial fauna and flora. Geol Mag 145: 173–197.

[10] Connor HE, Edgar E (1987) Name changes in the indigenous New Zealand flora, 1960–1986 and Nomina Nova IV, 1983–1986. New Zeal J Bot 25: 115–170.

[11] Heenan PB (1998a) Phylogenetic analysis of the *Carmichaelia* complex, *Clianthus*, and *Swainsona* (Fabaceae), from Australia and New Zealand. New Zeal J Bot 36: 21–40.

[12] Heenan PB (1998b) *Montigena* (Fabaceae), a new genus endemic to New Zealand. New Zeal J Bot 36: 41–51.

[13] Heenan PB (2000) *Clianthus* (Fabaceae) in New Zealand: a reappraisal of Colenso's taxonomy. New Zeal J Bot 38: 361–371.

[14] Wagstaff SJ, Heenan PB, Sanderson MJ (1999) Classification, origins and patterns of diversification in New Zealand Carmichaelinae (Fabaceae). Am J Bot 86: 1346–1356. 10487821

[15] Heenan PB, Dawson MI, Wagstaff SJ (2004) The relationship of *Sophora* sect. *Edwardsia* (Fabaceae) to *Sophora tomentosa*, the type species of the genus *Sophora*, observed from DNA sequence data and morphological characters. Bot J Linn Soc 146: 439–446.

[16] Weir BS, Turner SJ, Silvester WB, Park DC, Young JM (2004) Unexpectedly diverse *Mesorhizobium* strains and *Rhizobium leguminosarum* nodulate native legume genera of New Zealand, while introduced legume weeds are nodulated by *Bradyrhizobium* species. Appl Environ Microbiol 70: 5980–5987. 15466541 10.1128/AEM.70.10.5980-5987.2004 PMC522066

[17] Tang CKC, Koh WK, Bungard RA, Jones AV, Morton J, et al (2009) 16S rRNA characterisation of bacterial isolates from *Carmichaelia australis* and *C. corrugata*, New Zealand native legumes and assessment of their ability to produce functional nodules on five *Carmichaelia* species. Aspects Appl Biol 98: 203–206.

[18] Chen WX, Li GS, Qi YL, Wang ET, Yuan HL, et al (1991) *Rhizobium huakuii* sp. nov. isolated from the root nodules of *Astragalus sinicus* . Int J Syst Bacteriol 41: 275.280.

[19] Jarvis BDW, van Berkum P, Chen WX, Nour SM, Fernandez MP, et al (1997) Transfer of *Rhizobium loti*, *Rhizobium huakuii*, *Rhizobium ciceri*, *Rhizobium mediterraneum*, and *Rhizobium tianshanense* to *Mesorhizobium* gen. nov. Int J Syst Bacteriol 47: 895–898.

[20] Vincent JM (1970) A Manual for the Practical Study of Root-Nodule Bacteria. IBP Handbook 15. Oxford: Blackwell.

[21] Weisburg WG, Barns SM, Pelletier DA, Lane DJ (1991) 16S ribosomal DNA amplification for phylogenetic study. J Bacteriol 173: 697–703. 1987160 10.1128/jb.173.2.697-703.1991 PMC207061

[22] Young JM, Park D-C, Weir BS (2004) Diversity of 16S rDNA sequences of *Rhizobium* spp. implications for species determinations. FEMS Microbiol Lett 238: 125–131. 15336412 10.1016/j.femsle.2004.07.026

[23] Vinuesa P, Silva C, Werner D, Martínez-Romero E (2005) Population genetics and phylogenetic inference in bacterial molecular systematics: the roles of migration and recombination in *Bradyrhizobium* species cohesion and delineation. Mol Phylogenet Evol 34: 29–54. 15579380 10.1016/j.ympev.2004.08.020

[24] Turner SL, Young JPW (2000) The glutamine synthetases of rhizobia: Phylogenetics and evolutionary implications. Mol Biol Evol 17: 309–319. 10677854 10.1093/oxfordjournals.molbev.a026311

[25] Poly F, Ranjard L, Nazaret S, Gourbiére F, Monrozier LJ (2001) Comparison of nifH gene pools in soils and soil microenvironments with contrasting properties. Appl Environ Microbiol 67: 2255–2262. 11319109 10.1128/AEM.67.5.2255-2262.2001 PMC92864

[26] Laguerre G, Nour SM, Macheret V, Sanjuan J, Drouin P, et al (2001) Classification of rhizobia based on *nodC* and *nifH* gene analysis reveals a close phylogenetic relationship among *Phaseolus vulgaris* symbionts. Microbiol 147: 981–993. 10.1099/00221287-147-4-981 11283294

[27] Haukka K, Lindström K, Young JPW (1998) Three phylogenetic groups of *nodA* and *nifH* genes in *Sinorhizobium* and *Mesorhizobium* isolates from leguminous trees growing in Africa and Latin America. Appl Environ Microbiol 64: 419–426. 9464375 10.1128/aem.64.2.419-426.1998 PMC106060

[28] Zhang X-X, Turner SL, Guo X-W, Yang H-J, Debellé F, et al (2000) The common nodulation genes of *Astragalus sinicus* rhizobia are conserved despite chromosomal diversity. Appl Environ Microbiol 66: 2988–2995. 10877796 10.1128/aem.66.7.2988-2995.2000 PMC92101

[29] Tamura K, Peterson D, Peterson N, Stecher G, Nei M, et al (2011) MEGA5: Molecular evolutionary genetics analysis using maximum likelihood, evolutionary distance, and maximum parsimony methods. Mol Biol Evol 28: 2731–2739. 21546353 10.1093/molbev/msr121 PMC3203626

[30] Ampomah OY, Huss-Danell K (2011) Nodulation of *Thermopsis lupinoides* by a *Mesorhizobium huakuii* strain with a unique *nodA* gene in Kamtchatka, Russia. Appl Environ Microbiol 77: 5513–5516. 21652738 10.1128/AEM.00622-11 PMC3147426

[31] Cummings SP, Gyaneshwar P, Vinuesa P, Farrugia FT, Andrews M, et al (2009) Nodulation of *Sesbania* species by *Rhizobium* (*Agrobacterium*) strain IRBG74 and other rhizobia. Environ Microbiol 11: 2510–2525. 19555380 10.1111/j.1462-2920.2009.01975.x PMC7163632

[32] Weir BS (2006) Systematics, specificity and ecology of New Zealand rhizobia. PhD thesis. Auckland: The University of Auckland.

[33] Gu CT, Wang ET, Sui XH, Chen WF, Chen WX (2007) Diversity and geographical distribution of rhizobia associated with *Lespedeza* spp. in temperate and subtropical regions of China. Arch Microbiol 188: 355–365. 17530227 10.1007/s00203-007-0256-3

[34] Wang H, Man CX, Wang ET, Chen WX (2009) Diversity of rhizobia and interactions among the host legumes and rhizobial genotypes in an agricultural-forestry ecosystem. Plant Soil 314: 169–182.

[35] Sullivan JT, Patrick HN, Lowther WL, Scott DB, Ronson CW (1995) Nodulating strains of *Rhizobium loti* arise through chromosomal symbiotic gene transfer in the environment. Proc Natl Acad Sci USA 92: 8985–8989. 7568057 10.1073/pnas.92.19.8985 PMC41092

[36] Turner SL, Zhang XX, Li F-D, Young JPW (2002) What does a bacterial genome sequence represent? Mis-assignment of MAFF 303099 to the genospecies Mesorhizobium loti. Microbiol 148: 3330–3331. 10.1099/00221287-148-11-3330 12427922

[37] Kobayashi H, Broughton WJ (2008) Fine-tuning of symbiotic genes in rhizobia: flavonoid signal transduction cascade. In: Dilworth MJ, James EK, Sprent JI, Newton WE, editors. Nitrogen-fixing legume symbioses. Dordrecht: Springer. pp. 117–152.

[38] Rogel MA, Ormeño-Orrillo E, Martinez-Romero E (2011) Symbiovars in rhizobia reflect bacterial adaptation to legumes. Syst Appl Microbiol 34: 96–104. 21306854 10.1016/j.syapm.2010.11.015

[39] Lavin M, Herendeen PS, Wojciechowski MF (2005) Evolutionary rates analysis of Leguminosae implicates a rapid diversification of lineages during the Tertiary. Syst Biol 54: 575–594. 16085576 10.1080/10635150590947131

